# Medicine

**Published:** 1910-09

**Authors:** J. R. Charles


### Progress of the Medical Sciences.

### MEDICINE.

Further experiments have recently been made by Edmunds[1] with a view of ascertaining if there is any difference in function between the **thyroid and parathyroid glands.** These experiments tend to support the view previously held, that the functions of the parathyroid differ materially from those of the thyroid, and further, that parathyroids do not change into thyroid tissue even when left in animals in whom there is great thyroid insufficiency. Other experiments on his part confirm the view that if a large amount of calcium is given, or if the animals are fed mainly on milk, the symptoms following total thyroidectomy are mitigated, the percentage of recoveries after thyroidectomy being considerably increased.

Jean V. Cooke [2] has carried out a series of experiments on the excretion of calcium and magnesium after parathyroidectomy. The magnesium content in normal dogs' brains was found to be practically the same as in those of dogs dying with tetany, while the calcium content of the brains of two dogs dying with tetany was slightly higher than that of the control dogs. After parathyroidectomy the calcium excretion in the urine did not differ markedly from that of a fasting period, while there was some increase in the excretion of magnesium. In two animals with tetany an excretion of diacetic acid in the urine was found, a phenomenon which had previously been observed by Underhill and Saiki after thyro-parathyroidectomy with development of tetany. With reference to the magnesium and calcium content of the brain, no very definite conclusions can be drawn from the small number of cases studied, but these experiments do not support the contention of MacCallum that the parathyroids exert a very potent influence on calcium metabolism. The calcium and magnesium content of the fæces of normal and parathyroidectomised dogs in these experiments of Cooke was found to be similar.

The subject of **thyroid inadequacy** is dealt with in a paper by Leonard Williams, [3] in which he draws attention to the importance of the thyroid gland in fixing the calcium salts in the body. He maintains that the thyroid hypertrophies in pregnant women, and that the influence of its secretion may assist bone formation in the fœtus. In some women this leads to such exhaustion of the gland that they cannot suckle, lactation depending on a due supply of the thyroid secretion. He regards the bony phenomena of rickets as due to thyroid insufficiency. Calcium salts do not arrest the disease, because in the absence of sufficient thyroid secretion the ingested calcium is not assimilated by the bones. He has obtained good results in rickets by the administration of thyroid, a notable example being the case of one of twins (the other being quite healthy), both of whom had been fed and brought up in the same way.

Some cases of **nocturnal enuresis,** adenoids and enlarged tonsils he regards as due to thyroid inadequacy, and he points out that a large percentage of these follow infantile febrile diseases, which were the cause of the depressed junction of the thyroid. In such cases delayed puberty may yield to thyroid extract. Dysmenorrhœa may also be cured by thyroid administration, as may also migrainous attacks occurring during menstruation.

Léopold Lévi and H. de Rothschild attach considerable importance to what they call the eyebrow sign as evidence of thyroid inadequacy. This consists in the rarefaction or complete absence of the hair of the outer third of the eyebrow. Carious teeth and thin, delicate and friable nails are also symptoms. In their remarks on treatment, the authors state that thyroid extract acts very much better when administered in conjunction with iodine, arsenic, and calcium than by itself.

Some interesting observations and experiments on the so-called **thyroid carcinoma of the brook trout** and its relation to ordinary goitre have been made by D. Marine[4] and C. H. Lenhart. This disease is characterised by an abnormal and excessive growth of thyroid tissue which frequently forms visible tumours. Extreme degrees of the disease appear to be confined to the carnivorous fish reared in captivity. The observations were made in a large private hatchery. They regard the disease as one of endemic goitre and not as one of true cancer, giving ample reasons for this conclusion. Careful investigations were made into the conditions under which the fish of different ages lived. The authors were unable to find any grounds for regarding the disease as one of infectious origin. In this connection histological examinations have yielded negative results, and the fish will recover when placed in what is theoretically the most polluted water. On the other hand, the three factors of over-feeding, over-crowding, and limited water supply were considered to be directly concerned in the development of the disease, which they regarded as due to an abnormal stimulation of the thyroid, not by any single substance, whether the result of food decomposition or of excretory products, but as the result of the activity of a great variety of these. Iodine in the form of Lugol's solution added to the water in the trough effected cures. The same authors have carried out similar experiments on Lake Erie pike[5] with very similar findings. Carnivorous fish, like carnivorous mammals, are more frequently affected. Thus while changes are found in the pike they are absent in the carp. Stuart-Low[6] has removed the thyroid, or part of it, in cases of inoperative carcinoma, and reports a deterrent effect on the rate of growth of the primary tumour. The secondary glands also appear to be favourably influenced, becoming softer and less painful. In several cases the patient has put on weight, as much indeed as 15 lb. in five months being gained by a man who had previously been losing flesh.

REFERENCES.

1 *J. Path. and Bacteriol.*, 1910, xiv, 288.
2 *J. Exper. Med.*, 1910, xii, 45.
3 *Med. Rev.*, 1910, xiii, 13.
4 *J. Exper. Med.*, 1910, xii, 311.
5 *Johns Hopkins Hosp. Bull.*, 1910, xxi, 95.
6 *Lancet*, 1909, ii, 1138.

J. R. CHARLES.

